# Production of bioactive β-carotene by the endophytic bacterium *Citricoccus parietis* AUCs with multiple in vitro biological potentials

**DOI:** 10.1186/s12934-023-02108-z

**Published:** 2023-05-03

**Authors:** Noura Sh. A. Hagaggi, Usama M. Abdul-Raouf

**Affiliations:** Botany Department, Faculty of Science, Aswan University, Aswan, 81528 Egypt

**Keywords:** *Citricoccus parietis*, Carotenoids, Pigment, Antibacterial, Antioxidant, Antidiabetic

## Abstract

**Background:**

Although microalgae and plants are traditionally used for obtaining natural pigments, overexploitation and overharvesting threaten them. Bacteria represent a superior alternative for the production of pigments due to their ability to produce greater amounts in a short time without seasonal restrictions; furthermore, bacterial pigments have a wide range of uses and are safe and biodegradable. This study is the first on the production of ß-carotene as a promising bioactive agent from endophytic bacteria.

**Results:**

The yellow pigment produced by the endophytic bacterium *Citricoccus parietis* AUCs (NCBI accession number: OQ448507.1) was extracted by methanol and then purified and identified. One band was obtained by TLC analysis, which was identified as ß-carotene based on its spectroscopic and chromatographic characteristics. The pigment exhibited remarkable antibacterial, antioxidant and antidiabetic activities.

**Conclusions:**

This research may serve as a valuable starting point for exploiting *C. parietis* AUCs as a potent source of ß-carotene for biomedical therapies. To validate the findings of this research, in vivo studies must be performed.

## Background

Since the 1980s, synthetic pigments have been extensively used in numerous applications, including food, cosmetics, and pharmaceutical industries, but because of their harmful side effects, natural pigments are currently receiving greater attention due to their safety and environmentally friendly beneficial properties [1–3]. Although plant sources are widely employed for the extraction of natural pigments, seasonal variations have a direct impact on plant pigments, and widespread plant use puts valuable species at risk [4]. Microorganisms, on the other hand, have an advantage over plants in the synthesis and extraction of pigments since they develop quickly in affordable media regardless of the weather [5].

Carotenoids are among the pigments that are most commonly employed in human nutrition and health. These are isoprenoid macromolecules that are produced in a variety of species, such as plants, algae, fungi, and some bacteria [6]. Carotenoids have a wide range of biological functions, including antioxidant, anti-inflammatory, antibacterial, antidiabetic, and anti-cancer characteristics, and they are used in a wide range of products, including food, feed, medications, and cosmetics [7].

Carotenoids are essential for bacteria because they shield them from UV light, reduce oxidative stress, and keep the cell membrane flexible at low temperatures [8]. The most promising alternative for the synthesis of natural carotenoids is bacteria because of their numerous unique features, such as quick and simple growth using affordable culture media, control over the conditions of fermentation, and the potential for genetic modification [9]. In order to maximize the production and extraction yield of novel carotenoids from bacteria and to commercialize bacterial carotenoids, efforts should be increased in this direction.

Although some studies have reported carotenoids-producing bacteria from soils and marine environments [10], according to our knowledge no research on the production and biological activities of carotenoids from endophytic bacteria. Considering the above facts, this study aimed to extract, characterize, and evaluate the expected in vitro biological potentials of the yellow pigment produced by the endophytic bacterium *Citricoccus parietis* AUCs.

## Materials and methods

**Fig.1:**
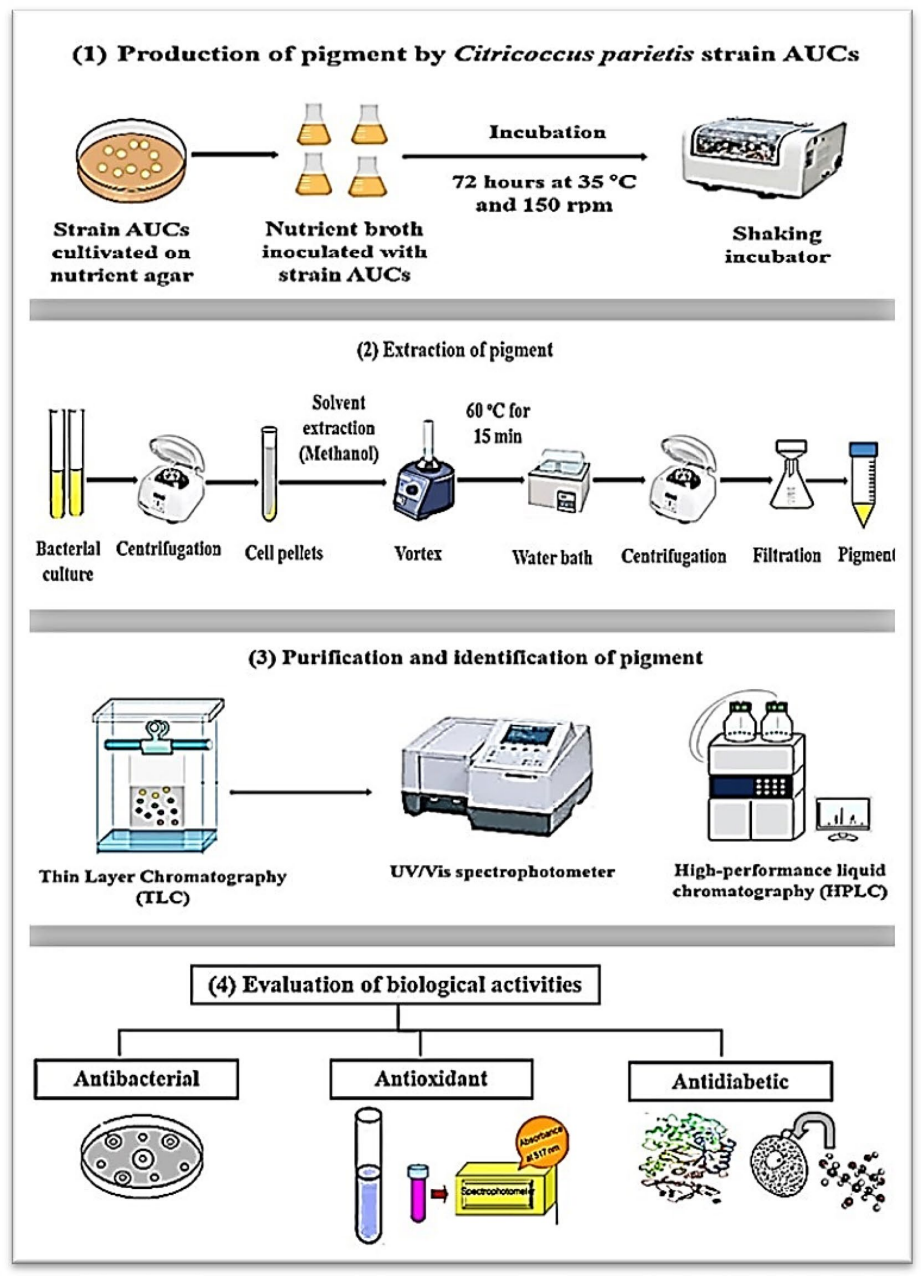
Experimental design showing the steps of the study, including pigment production by *C. parietis* AUCs, extraction, purification, identification, and evaluation of its in vitro biological activities

The experimental design included in this study is summarized in Fig. 1. Three replicas were made for each experiment. The values were expressed as the means of three replicas ± standard errors.

### Bacterial strain

**Fig.2:**
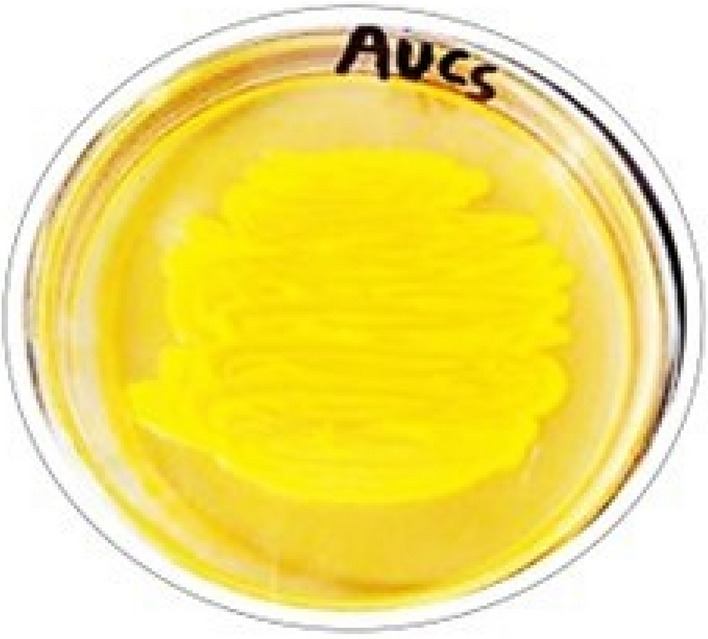
The endophytic bacterium *Citricoccus parietis* AUCs grown on nutrient agar

*Citricoccus parietis* strain AUCs (accession number: OQ448507.1) is a yellow pigmented endophytic bacterium (Fig. 2) previously isolated by us from the stem of the medicinal plant *Calotropis procera*.

### Production and extraction of pigment

Strain AUCs was grown in nutrient broth for 72 h at 35 °C and a shaking rate of 150 rpm. Pigment extraction was performed following the procedure described by Ezhil et al. [11] with slight modification. Bacterial cultures were centrifuged, and pigmented biomass pellets were collected. Methanol was mixed with bacterial pellets in a ratio of 2:1 (solvent/pellets, v/w), vortexed for 1 min and then incubated in water bath at 60 °C for 15 min. The yellow colored supernatant was centrifuged and filtered. Solvent was evaporated, the extracted yellow pigment was weighted, and the yield of pigment production was evaluated as µg per gram of cell biomass. The pigment extract was then stored in darkness at 4 °C for further study.

### Pigment purification

The crude yellow pigment (1 mg) was re-dissolved in 10 ml methanol and then subjected to Thin Layer Chromatography (TLC) analysis according to Kusmita et al. [12]. On aluminum TLC plates (Silica Gel 60 F254, Merck® Darmstadt, Germany), the pigment extract was spotted along with ß-carotene (Sigma-Aldrich) as a standard. The plates were developed in a mixture of acetone: n-hexane (6:4 v/v). The yellow band was scraped off from the silica plates. The retention factor (Rf) was calculated according to Stahl [13].

### Pigment identification

The scraped yellow bands obtained from TLC analysis were eluted with methanol and subjected to ultraviolet–visible spectrophotometer and high-performance liquid chromatography- diode array detector (HPLC–DAD) analysis. The spectra of the yellow pigment were recorded at wavelengths of 350–600 nm by a T60U UV–Vis spectrophotometer (PG Instruments Ltd). The data were compared with those reported by previous studies as well as ß-carotene (Sigma-Aldrich) as a standard [14].

Twenty microliter of the extract was injected onto an Hypersil ODS (C18) column (250 × 4.6 mm, 5 µm; Thermo Scientific). The column was eluted with acetonitrile and methanol (10:90 v/v) at a flow rate of 1.5 ml/min and 30 °C. Detection was carried out by diode array detector at 400–500 nm [15]. The obtained results were compared with standard ß-carotene (Sigma-Aldrich).

### Optimization of pigment production

One-factor-at-a-time method [16] was used to determine the optimum parameters of pigment production by *C. parietis* AUCs. The effect of different parameters *i.e.,* culture media (nutrient broth, trypticase soy broth, and potato dextrose broth), pH values (5, 6, 7, 8, 9 and 10), temperatures (10, 15, 20, 25,30, 35 and 40 °C), incubation periods (24, 48, 72, 96 and 120 h) and shaking rates (0 (static), 50, 100, 150 and 200 rpm) on the pigment production was evaluated. Growth and pigment production were measured spectrophotometrically at 600 and 450 nm, respectively. Pigment concentrations were calculated using ß-carotene standard curve.

### Growth and pigment production kinetics

Growth kinetics along with pigment production kinetics were determined by growing *C. parietis* AUCs in nutrient broth at optimized conditions. Culture samples were collected every 10 h, and the growth was measured at 600 nm. Culture samples were then centrifuged, the pigment was extracted from the cell biomass, and it was weighed. Growth and pigment production were plotted against time.

### In vitro antibacterial activity of *C. parietis* AUCs pigment

Four pathogenic bacteria, *i.e.,* Gram positive (*Staphylococcus aureus* ATCC 25923 and *Streptococcus agalactiae* ATCC 13813) and Gram negative (*Pseudomonas aeruginosa* ATCC 9027 and *Klebsiella pneumonia* ATCC 4352), were used to evaluate the antibacterial activity of the pigment produced by *C. parietis* AUCs by agar well-diffusion following the Clinical and Laboratory Standards Institute (CLSI) [17]. These bacteria were obtained from the stock cultures of the bacteriology lab, Botany Department, Faculty of Science, Aswan University, by Prof. Dr. Usama Abdul-Raouf (co-author), the head of the bacteriology lab. Pigment extract (1 mg/mL in methanol) was introduced into wells (6 mm) pierced in nutrient agar plates that were inoculated with 100 µL of bacterial suspension (10^7^ CFU/mL). The positive and negative controls were ampicillin solution (1 mg/mL) and methanol, respectively. The diameters of inhibition zones that appeared around the wells after 48-h of incubation at 35 °C were measured in millimeters.

### In vitro antioxidant activity of *C. parietis* AUCs pigment

#### Total antioxidant activity

Total antioxidant activity of the pigment extract was evaluated using Phosphomolybdenum assay [18]. In a test tube, 1 mL of reagent solution (0.6 M H_2_SO_4_, 28 mM Na_2_HPO_4_ and 4 mM (NH_4_)_6_Mo_7_O_24_) was mixed with 1 mL of pigment extract (1 mg/mL). The reaction mixture was incubated in a water bath at 95 ± 2 °C for 90 min. Methanol and ascorbic acid were used as negative control and standard antioxidant compound, respectively. Absorbance was measured at 695 nm by a T60U UV/Vis spectrophotometer.

#### Free radical scavenging activity

The pigment's ability to scavenge free radicals was estimated using diphenyl picrylhydrazyl (DPPH) following the procedure of Jimoh et al. [19]. Briefly, 100 µL of fresh DPPH reagent prepared in methanol (0.1 mM) was mixed with 100 µL of pigment extract (1 mg/mL in methanol). Methanol was used as a control. Reaction was carried out in the darkness at the room temperature for 30 min. Absorbance was read at 517 nm and the percentage of scavenging activity was calculated as follows:

$$ {\text{Scavenging activity }}\left( {\text{\% }} \right) = \frac{{{\text{Absorbance of control}} - {\text{Absorbance of pigment }}}}{{\text{Absorbance of control}}} \times 100 $$

### In vitro antidiabetic activity of *C. parietis* AUCs pigment

#### α-Amylase inhibitory assay

The ability of *C. parietis* AUCs pigment to inhibit pancreatic α-amylase activity was determined according to the method of Sudha et al. [20]. Porcine pancreatic α-amylase (Sigma-Aldrich) solution (1 unit/mL) and starch solution (0.5%) were immediately prepared in phosphate buffer (20 mM, pH 6.9). In a test tube, 200 µL of α-amylase solution was added to 200 µL of the pigment extract and incubated for 15 min at 37 °C. Then, 200 µL of starch solution was added and the reaction mixture was further incubated at 37 °C for 15 min. Tubes containing an identical mixture without the pigment extract served as a control. The reaction was stopped by adding 200 µL of dinitrosalicylic acid reagent. Tubes were boiled in a water bath for 10 min and then cooled. The absorbance was measured at 540 nm. The percentage of α-amylase inhibition was calculated using the following equation:

$$ {\upalpha }\,{ - }\,{\text{amylase inhibition }}\left( {\text{\% }} \right) = { }\frac{{{\text{Absorbance of control}} - {\text{Absorbance of pigment}}}}{{\text{Absorbance of control}}} \times 100 $$

#### Glucose uptake by yeast cell model

Yeast cells were used as a model to evaluate the effect of the present pigment on the efficiency of glucose uptake. The assay was performed following the method of Pulivarthi et al. [21]. The suspension (10%, w/v) was prepared from commercial baker’s yeast (*Saccharomyces cerevisiae*) and was set overnight at 25 °C. The suspension was then centrifuged many times until it became clear. The pigment extract (1 mg/mL) was mixed with 1 mL of glucose solutions (5 mM and 10 mM) and incubated at 37 °C for 10 min. Then, 100 µL of yeast suspension was added, vortexed, and incubated at 37 °C for 60 min. The mixture was centrifuged, and the glucose content of the supernatant was estimated spectrophotometrically at 520 nm. Tubes containing all the contents except the pigment extract were used as controls, and metformin (1 mg/mL) was used as an antidiabetic drug. Glucose uptake (%) was calculated as follows:

$$ {\text{Glucose uptake }}\left( {\text{\% }} \right) = \frac{{{\text{Absorbance of control}} - {\text{Absorbance of sample}}}}{{\text{Absorbance of control}}} \times 100 $$

## Results and discussion

### Pigment production, purification and identification

**Fig.3:**
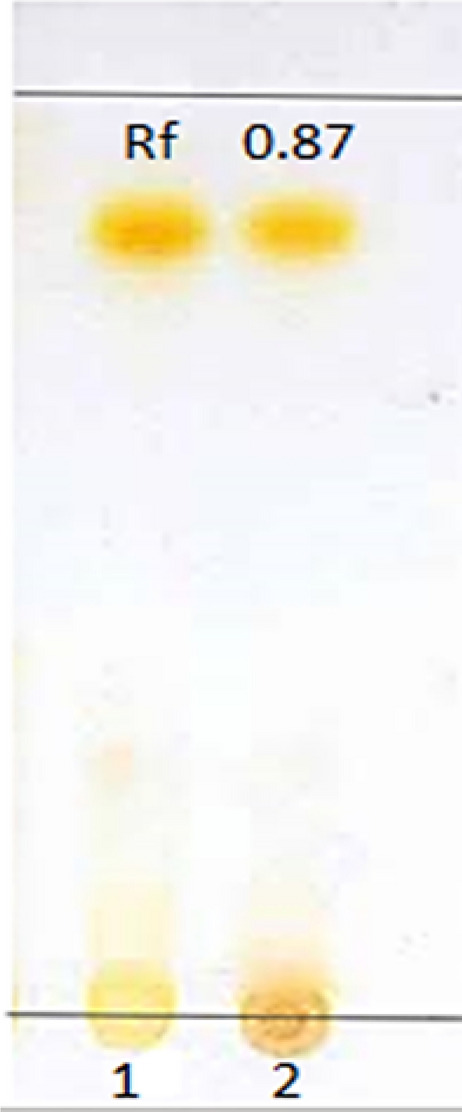
Thin-Layer Chromatography (TLC) of *C. parietis* AUCs yellow pigment and standard ß-carotene using acetone and n-hexane in a 6:4 (v/v) ratio shows bands with an Rf value of 0.87. 1 is the pigment extract and 2 is the standard ß-carotene

**Fig.4:**
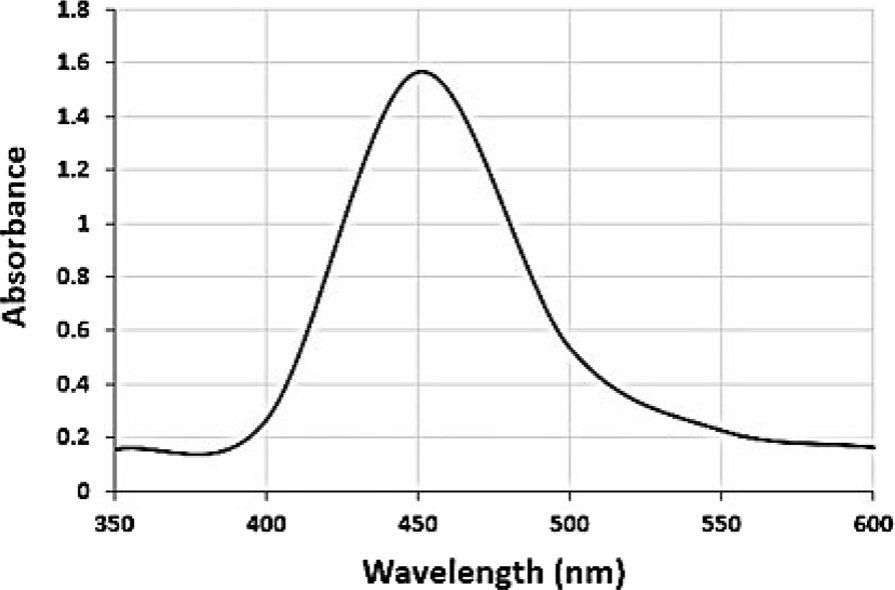
UV–Vis spectra of *C. parietis* AUCs yellow pigment

**Fig.5:**
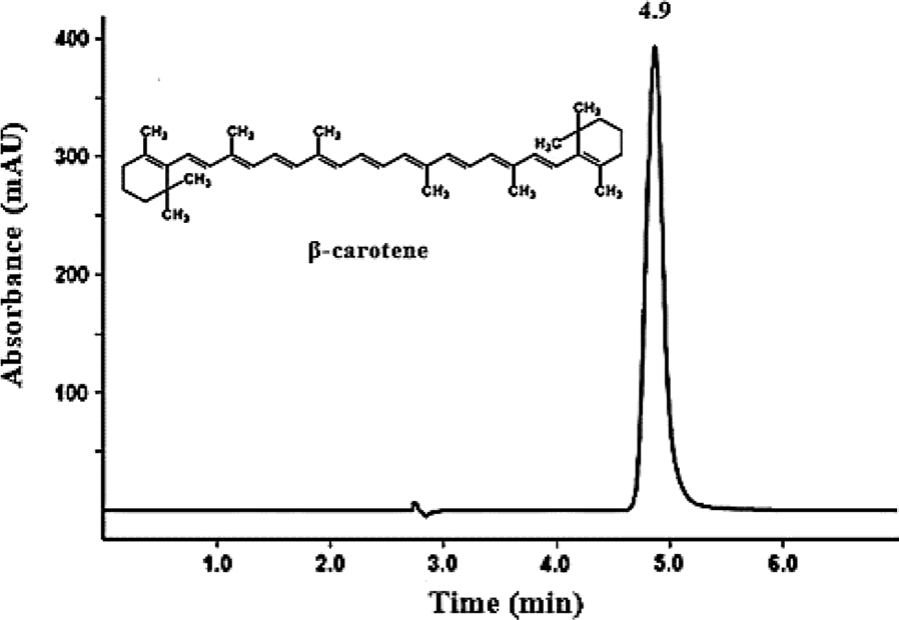
HPLC chromatogram of *C. parietis* AUCs yellow pigment

A yellow pigment was produced by the endophytic bacterium *C. parietis* AUCs with a yield of 491.6 ± 5.5 µg/g biomass. The pigment was separated on TLC plates using a mixture of acetone and n-hexane at a 6:4 ratio. One yellow band was observed with an Rf value of 0.87 (Fig. 3). The same Rf value was observed previously for ß-carotene of *Micrococcus roseus* [22], which indicated that the yellow pigment of *C. parietis* AUCs may be ß-carotene. Furthermore, the spectroscopic and chromatographic characteristics of the pigment were determined using UV/Vis spectrophotometer and HPLC–DAD. The UV/Vis spectra of the pigment extract were detected at wavelengths of 350–600 nm. The maximum peak was at 450 nm (Fig. 4), which indicated the presence of carotenoid compound [23]. Our finding was in accordance with those of Dawoud et al. [24] and Naz et al. [25], who reported that the maximum absorption of yellow pigment produced by *Bacillus* sp. DBS4 and *Mucor circinelloides* was at 450 nm. The HPLC–DAD analysis revealed one peak at 4.9 min, which was identified as β-carotene (Fig. 5).

### Optimization of pigment production

**Fig.6:**
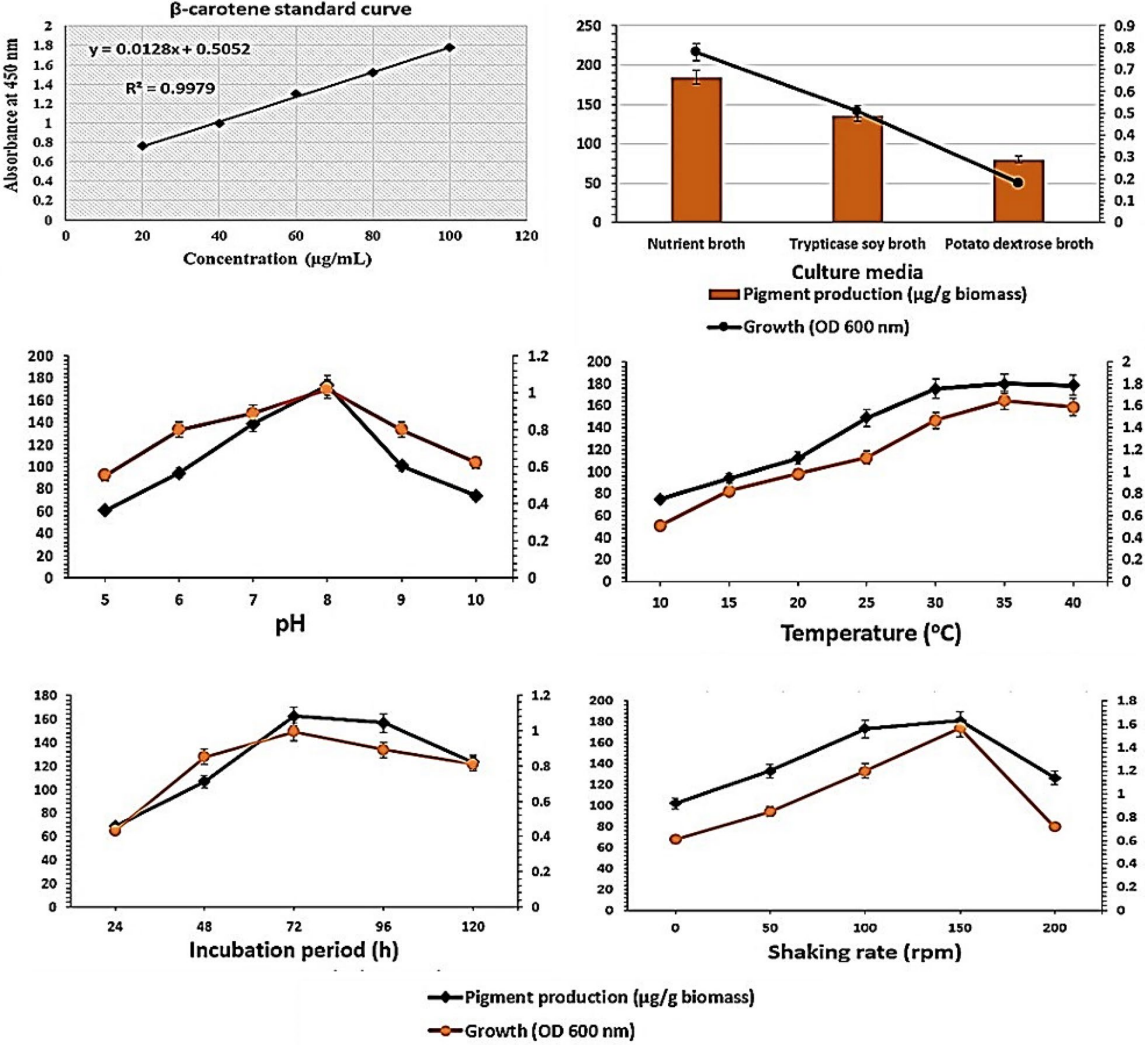
Optimization of pigment production by *C. parietis* AUCs

The growth of bacteria and the biosynthesis pathways of their pigments are directly affected by culture conditions and environmental parameters [24]. In this study, the effects of different parameters *i.e.,* culture media, pHs, temperatures, incubation periods and shaking rates on the growth and the production of pigment by *C. parietis* AUCs were investigated. It was noticed that the highest production of the pigment occurred under the same conditions that achieved optimum growth (Fig. 6). The optimum growth and pigment production by *C. parietis* AUCs were achieved in nutrient broth at pH 8, 35 °C after 72 h under 150 rpm. Many researchers have reported that the production of bacterial pigments is influenced by culture and environmental conditions [10, 16, 17].

### Growth and pigment production kinetics

**Fig.7:**
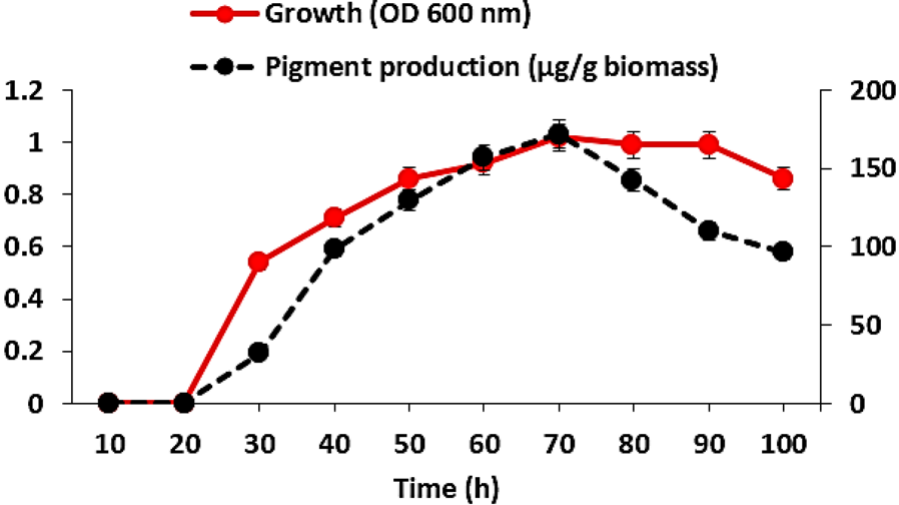
Growth and pigment production kinetics of *C. parietis* AUCs

The growth and pigment production kinetics for *C. parietis* AUCs were shown in Fig. 7. It was observed that pigment production was associated with cell growth. The exponential (log) phase for *C. parietis* AUCs started after 10 h of cultivation and continued up to 70 h. Then, the stationary phase takes about 20 h, after which the growth declined. The pigment production started after 30 h, and the highest production was gained at the end of the exponential phase at 70 h.

### In vitro antibacterial activity of pigment extract

**Fig.8:**
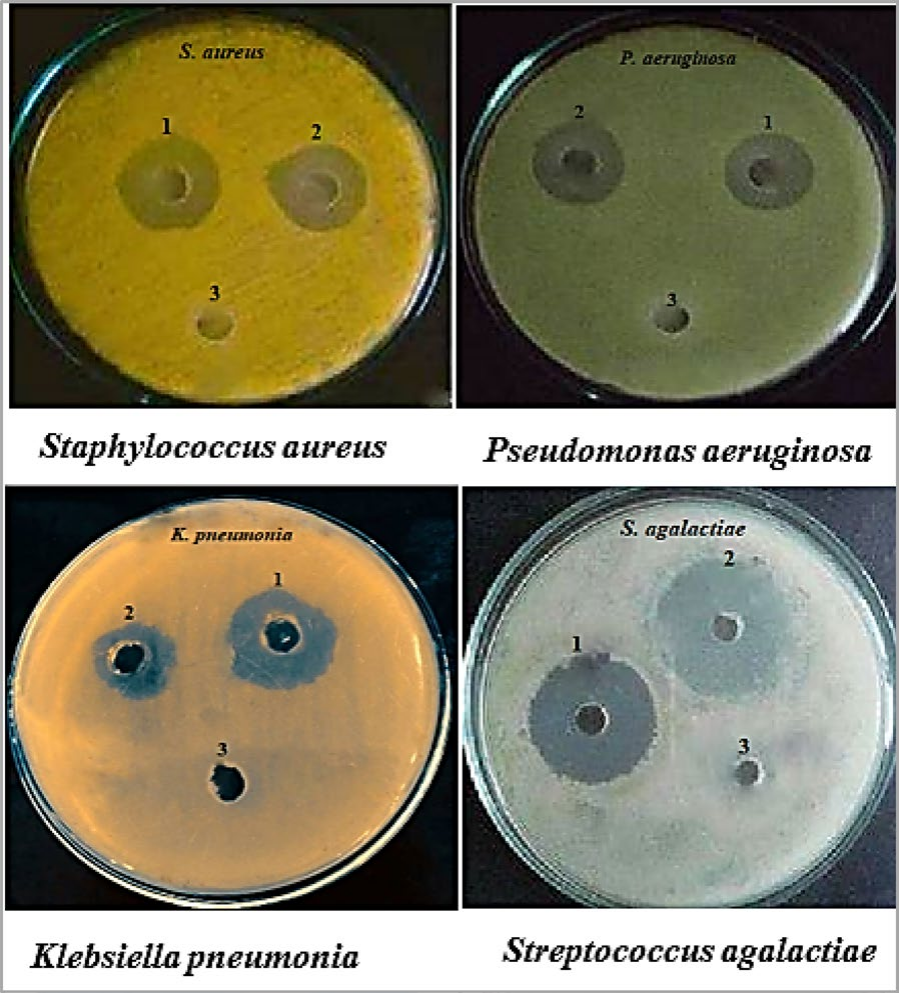
Antibacterial activity of *C. parietis* AUCs pigment extract. 1: Positive control (ampicillin solution, 1 mg/mL) 2: Pigment extract (1 mg/mL in methanol), and 3: Negative control (methanol)

As shown in Table 1 and Fig. 8, the pigment extract exhibited antibacterial activity against all of the tested pathogenic bacteria, with varying inhibition zone diameters based on bacterial species. The antibacterial activities of bacterial carotenoids extracted from *Micrococcus* sp., *Bacillus* sp., *Kocuria* sp., *Brevibacterium* sp. and *Virgibacillus* sp. were previously reported [16, 26, 27]. The ability of carotenoids to inhibit bacteria may be due to their ability to interact with proteins located in the outer membranes of bacterial cells, causing membrane damage that restricts the availability of nutrients needed for bacterial growth and, ultimately, results in bacterial death [28, 29].

**Table 1:** Antibacterial activity of *C. parietis* AUCs pigment extract

Pathogenic bacteria	Inhibition zone (mm)		
	Positive control (ampicillin solution, 1 mg/mL)	Pigment extract (1 mg/mL in methanol)	Negative control (methanol)
*Staphylococcus aureus*	21 ± 1.5	19 ± 0.9	0.0 ± 0
*Pseudomonas aeruginosa*	18 ± 1.2	20 ± 1.5	0.0 ± 0
*Klebsiella pneumonia*	22 ± 1.5	17 ± 0.9	0.0 ± 0
*Streptococcus agalactiae*	25 ± 1.7	27 ± 1.5	0.0 ± 0

Values are the means of three replicas ± standard errors

### In vitro antioxidant activity of pigment extract

The pigment extract of *C. parietis* AUCs had considerable total antioxidant activity (3097.5 ± 5.4 µg ascorbic acid equivalent/mg pigment extract) and DPPH scavenging activity (87%). The ability of microbial extracts to scavenge DPPH radicals was previously documented by other researchers [30]. Our findings corroborated those of other studies, which demonstrated that the carotenoids from bacteria such as *Kocuira marina* DAGII, *Pedobacter* sp., *Staphylococcus aureus*, and *Fontibacter flavus* YUAB-SR-25 showed strong antioxidant properties [31–33]. Carotenoids are recognized as antioxidant agents due to their triplet state energy and their electron-rich polyene structure [34]. Carotenoids can scavenge reactive oxygen species (ROS) through transferring electrons, oxidation, or forming carotenoid-radical cations, as well as they can prevent the generation of radicals by deactivating the electronically excited sensitizer molecules [35, 36].

### In vitro antidiabetic activity of pigment extract

One of the main strategies for managing diabetes is the inhibition of the activity of pancreatic α-amylase, which is the main enzyme involved in the breakdown of dietary starch into glucose [37, 38]. In the present study the pigment extract inhibited the activity of pancreatic α-amylase by 73.8%. The inhibitory effect of carotenoids against pancreatic α-amylase was reported by previous studies [39].

Despite the fact that yeast cells are different from human ones, glucose transport through the yeast cell membrane has gained attention as an in vitro testing method for antidiabetic activity [40]. Interestingly, the pigment extract of *C. parietis* AUCs increased the uptake of glucose by the yeast cells in a manner proportional to the concentration of glucose, where the uptake percentage increased with glucose concentration. The pigment extract increased the glucose uptake percentage by the yeast cells by 55.32 and 71.7% at 5 mM and 10 mM glucose concentrations, respectively. The antidiabetic effect of the pigment extract may be related to its antioxidant properties, as reported for other carotenoids [41, 42]. Several studies proved that carotenoids could lower plasma glucose levels and insulin resistance in humans, which consequently reduced diabetes risk [43]. Finally, this study sheds light on bacterial ß-carotene, which has proven efficacy as an antibacterial, antioxidant, and antidiabetic agent, making it a promising source for biomedical applications after conducting in vivo studies and ensuring the safety of its use.

## Conclusion

In this study, a yellow pigment was extracted from the endophytic bacterium *Citricoccus parietis* AUCs. The pigment was purified and characterized using TLC, UV/Vis spectrophotometer, and HPLC–DAD. Results revealed that the pigment is β-carotene. The production of pigment by *C. parietis* AUCs was optimized, and the biological activities of the pigment extract were evaluated in vitro. The study’s findings showed that *C. parietis* AUCs’ pigment has strong antibacterial, antioxidant, and antidiabetic properties. To exploit the results of this study in medical and pharmaceutical applications, in vivo experiments must be performed in order to validate our findings.

## Data Availability

The dataset supporting the conclusions of this article are available in the [NCBI] repository [https://www.ncbi.nlm.nih.gov/nuccore/OQ448507.1/].
